# Newly isolated lactic acid bacteria from silage targeting biofilms of foodborne pathogens during milk fermentation

**DOI:** 10.1186/s12866-019-1618-0

**Published:** 2019-11-08

**Authors:** Elizaveta Gavrilova, Elizaveta Anisimova, Alsu Gabdelkhadieva, Elena Nikitina, Adel Vafina, Dina Yarullina, Mikhail Bogachev, Airat Kayumov

**Affiliations:** 1 Kazan Federal University, 18 Kremlevskaya Str, 420008 Kazan, Russia; 2 Kazan National Research Technological University, 68 Karl Marx Str, 420015 Kazan, Russia; 3 Saint-Petersburg Electrotechnical University, 5 Professor Popov str, 197376 St. Petersburg, Russia

**Keywords:** Lactic acid bacteria, *Lactobacillus*, Antagonism, Biofilms, Milk fermentation

## Abstract

**Background:**

Raw milk, meat and plant materials are subjected to high risks of contamination by various pathogenic bacteria and thus their growth prevention is a great challenge in the food industry. Food fermentation by lactic acid bacteria (LAB) besides changing its organoleptic characteristics also helps to eliminate unfavorable microflora and represses growth of pathogens. To the date only few LABs has been reported to exhibit activity against bacteria embedded in the biofilms characterized by extreme resistance to antimicrobials, high exchange rate with resistance genes and represent high risk factor for foodborne disease development.

**Results:**

Six novel LAB strains isolated from the clover silage exhibited pronounced antibacterial activity against biofilm embedded pathogens. We show explicitly that these strains demonstrate high acidification rate, completely repress the growth of *E. coli*, *S. aureus* and to a lesser extent *P. aeruginosa* as well as exhibit appropriate probiotic and milk-fermenting properties. Moreover, in contrast to the approved probiotic strain *Lactobacillus plantarum* 8PA3, the new isolates were able to efficiently eradicate preformed biofilms of these pathogens and prevent bacterial spreading originating from the biofilm. We suggest these strains as potential additives to the pre-cultures of conventional LAB strains as efficient tools targeting foodborne pathogens in order to prevent food contamination from either seeded raw material or biofilm-fouled equipment.

**Conclusions:**

The AG10 strain identified as *L. plantarum* demonstrate attractive probiotic and milk fermentation properties as well as high resistance to simulated gastric conditions thus appearing perspective as a starter culture for the prevention of bacterial contamination originating from fouled equipment during milk fermentation.

## Background

Fermented milk products constitute significant part of the human nutrition with their quality and safety largely depending on both the milk itself and the starter cultures used for its fermentation. During fermentation process, Lactic acid bacteria (LAB) produce various metabolites thereby changing organoleptic characteristics of the substrates. In particular, fermentation of substrates improves digestibility and nutritional quality of the final product, enriches it with vitamins, essential amino acids and fatty acids [1].

Another important role of LAB is the prevention of pathogenic microorganisms growth thus reducing the risks of foodborne disease development (for a detailed review, we refer to [2–5]). The raw (unpasteurized) milk, meat and other raw materials often contain a variety of pathogenic bacteria which should be eliminated during the fermentation [6, 7]. Thus, various LAB metabolites like organic acids, hydrogen peroxide and bacteriocins act as bio-preservative agents thereby improving food safety and extending the storage period of the final product [2]. Therefore the growth, acidification rate and antimicrobial activity are the most important properties of newly isolated strains as potential candidates for biotechnological and food industry applications [8–10].

The antagonistic properties of LAB seem to be attractive also for targeting pathogens in biofilm embedded forms [11–14]. Biofilm is a complex three-dimensional microbial consortium where bacteria are embedded in an extracellular matrix of organic polymers produced by bacteria themselves [15–17]. The matrix drastically reduces the susceptibility of bacteria to different outer stress factors [18] providing up to 1000-fold higher tolerance to antimicrobials of the biofilm-embedded cells compared to their planktonic counterparts [19–22]. Recent data indicates that LAB efficiently prevent the biofilm formation as well as exterminate biofilm-embedded pathogens like *Staphylococcus epidermidis, Staphylococcus aureus, Citrobacter freundii, Enterobacter cloaceae, Klebsiella oxytoca, Proteus mirabilis* and *Candida albicans* [23–29]..

The spectrum of metabolites produced by LAB is strongly strain-specific, and thus the appropriate choice of the starter bacterial strain with specific characteristics determines the final product properties and quality [30–33]. Given the great variety of properties manifested by different strains, screening of novel LABs exhibiting attractive biological and technological properties until now remains one of key research directions in food microbiology.

Lactic acid bacteria are widely distributed in natural fermented foods as indigenous microflora [34] making such products a common source of lactic acid bacteria with potentially interesting functional and technological properties as well as perspective probiotics. Although traditionally LAB stains were isolated from the raw milk of various animals and naturally fermented dairy products [35–38], in recent years there is an increased interest to LAB strains with potential probiotic properties from unconventional sources like fecal samples, soil and fruits, especially as a part of dietary for subjects with lactose intolerance (for a detailed review, we refer to [39]). Despite increasing interest, to the date only few studies described the isolation of indigenous LAB stains from the silage [40–42] indicate that quite little information is available regarding their microbial ecology and thus also about their potential usefulness for dairy production.

Here we report the isolation and characterization of LABs from clover silage produced in the Republic of Tatarstan, Russia, exhibiting high anti-biofilm activity and probiotic properties.

## Results

### Antibacterial activity of LAB

From the 5-month fermented clover silage, the natural habitat of LAB, 120 catalase-negative isolates forming the highest halo zones on CaCO_3_-containing plates were selected and subjected to further screening for antibacterial activity. Out of the 120 colonies initially subjected to screening, six isolates exhibiting the most pronounced antagonistic properties against various pathogenic bacteria in agar diffusion test were selected (Table 1).

**Table 1:** Antimicrobial activity of LAB strains isolated from silage (agar drop diffusion test)

LAB species	Strain	Growth inhibition, mm				
		*Escherichia coli* MG1655 (K-12)	*Klebsiella pneumonia (Clinical isolate)*	*Pseudomonas aeruginosa* АТСС 27853	*Bacillus cereus (Clinical isolate)*	*Staphylococcus aureus subsp. aureus* ATCC 29213
*Lactobacillus plantarum*	AG1	8.5 ± 2.1	10.0 ± 2.8*	13.0 ± 2.8*	5.5 ± 2.1	9.0 ± 1.4
*Lactobacillus fermentum*	AG8	8.5 ± 2.1	9.5 ± 0.7	13.0 ± 2.8*	6.5 ± 0.7*	9.5 ± 0.7*
*Lactobacillus plantarum*	AG9	8.5 ± 2.1	8.5 ± 2.1	8.0 ± 2.8	6.0 ± 0.3	7.5 ± 2.1
*Lactobacillus plantarum*	AG10	8.0 ± 1.4	11.0 ± 1.4*	11.0 ± 1.4*	6.0 ± 1.4	9.5 ± 0.7*
*Lactobacillus plantarum*	AG15	10.0 ± 0.2*	11.5 ± 0.7*	12.5 ± 3.5*	6.5 ± 0.7*	9.0 ± 0.2
*Lactobacillus fermentum*	AG16	9.0 ± 1.4	13.0 ± 0.2*	12.5 ± 3.5*	7.0 ± 0.2*	9.5 ± 2.1*
*Lactobacillus plantarum*	8PA3	7.5 ± 1.6	7.9 ± 0.6	9.1 ± 0.4	5.0 ± 0.5	8.0 ± 1.3

* denotes statistically significant difference with probiotic strain *L. plantarum* 8PA3 (*p* < 0.05)

**Fig. 1:**
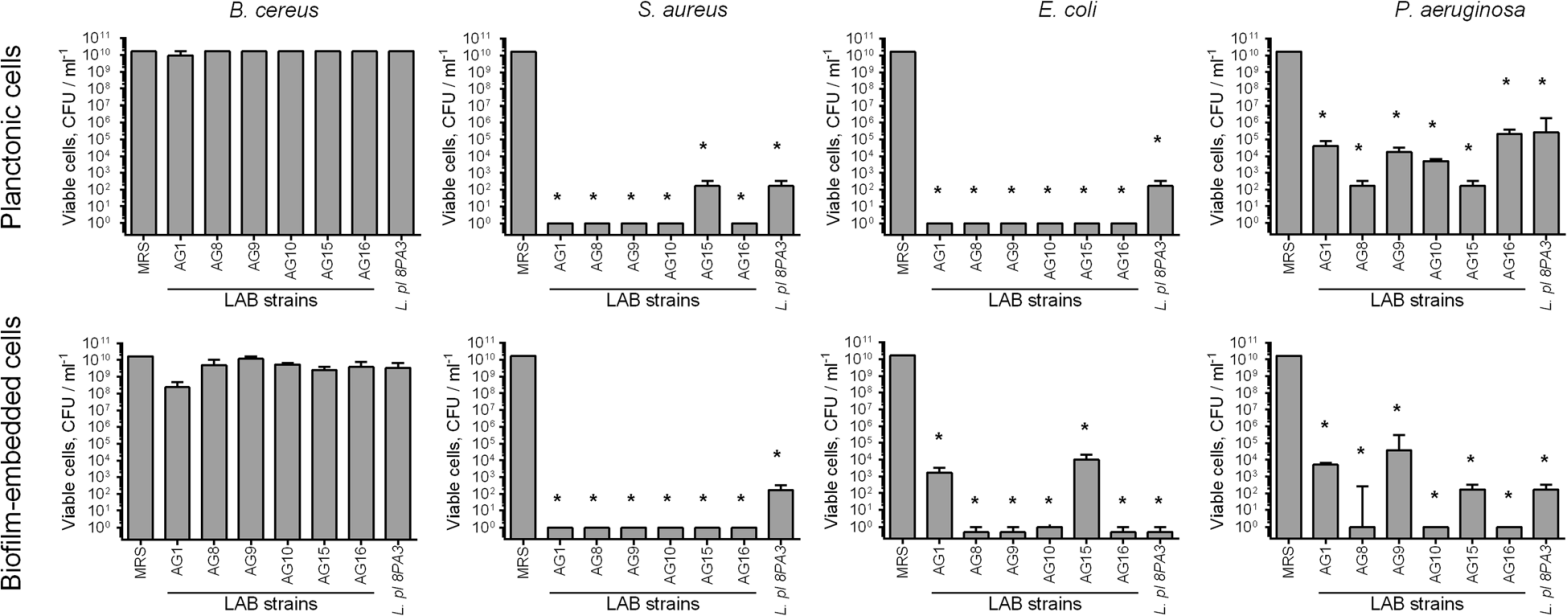
Antimicrobial activity of novel LAB strains against planktonic and biofilm embedded forms of pathogenic bacteria. LAB suspensions in MRS broth without sorbic acid (107 CFU/ml) were added to 48-h old biofilms of pathogenic bacteria. After 24 h CFUs of bacteria were calculated on differential media. Data are shown as medians with IQR. Asterisks denote statistically significant difference with monocultures of pathogenic bacteria (*p* < 0.05)

For all selected LAB strains with the exception of AG9 zones of inhibition of pathogens growth were significantly higher or comparable with those of the industrial probiotic strain *L. plantarum* 8PA3. Since the activity against bacteria embedded in biofilms has been previously reported only for few LAB, the biofilm-eradicating activity of these novel LAB strains was tested explicitly. For that, the suspensions of LAB (10^7^ CFU/mL) in MRS broth were added to the wells with 48-h old biofilms pre-formed by pathogenic bacteria washed with sterile saline to simulate the contaminated/biofouled surface, and the incubation was continued for the next 24 h. Then the CFUs numbers of pathogens and LAB have been differentially counted by using differential media in the culture liquid (Fig. 1, upper lane) and in the residual biofilm (Fig. 1, lower lane). No repression of *B. cereus* spreading from the biofilm and consequent growth could be observed in the presence of any of the LAB. On the other hand, no viable *S. aureus* cells were detected in both culture liquid and biofilm after 24 h of co-cultivation with any of the LAB strains with the exception of AG15. Remarkably, AG16 and AG10 strains led to almost complete eradication of both *P. aeruginosa* and *E. coli* in the biofilm. Of note, the probiotic strain *L. plantarum* 8PA3 exhibited the lowest rate of the pathogens growth repression in both culture liquid and biofilms.

CFUs number of LAB themselves in all mixed cultures insignificantly decreased in comparison with monocultures and remained within the range 10^8^–10^10^ CFU/mL, while in the presence of *P. aeruginosa* CFUs number of AG9, AG10, AG15 and AG16 decreased by 3–4 orders of magnitude (Additional file 1: Fig. S1), apparently, as a consequence of strong antagonistic interactions of *P. aeruginosa* with many other bacteria including LAB [43].

**Fig. 2:**
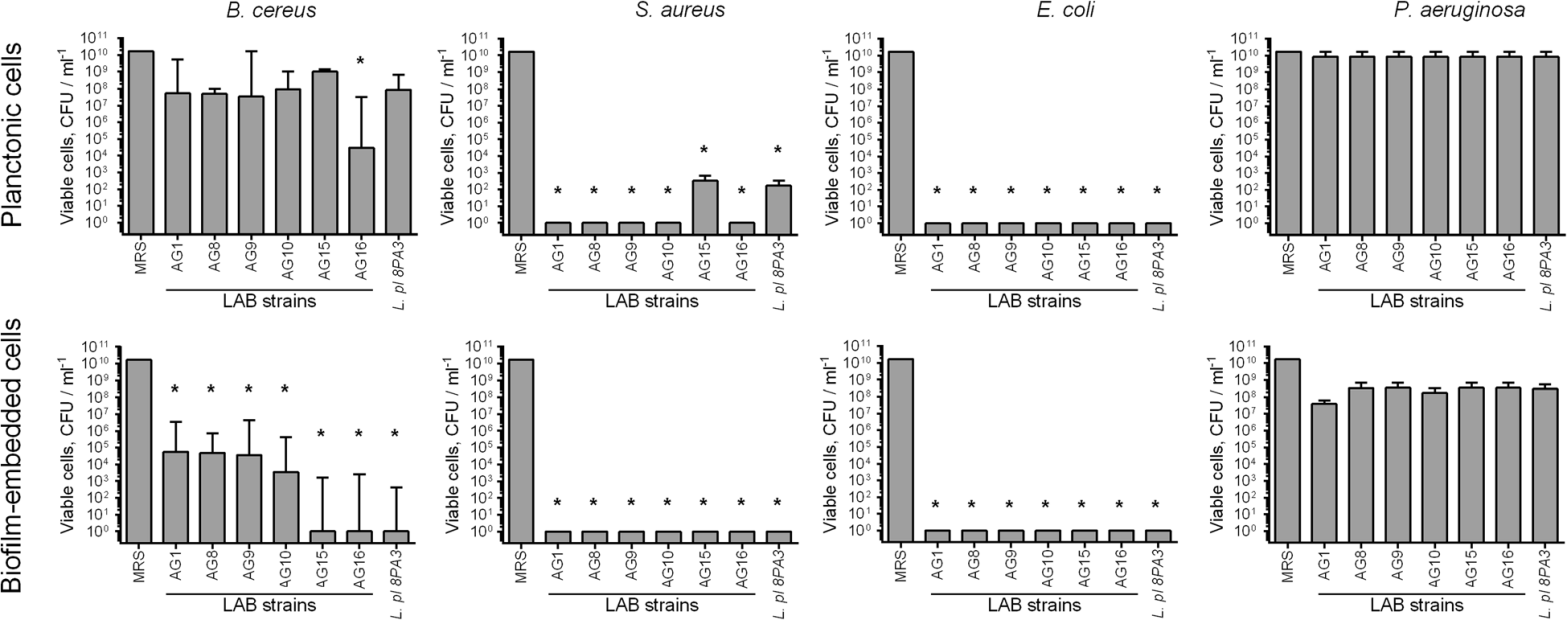
Repression of pathogenic bacteria by newly isolated LAB strains in co-cultivation experiments. Suspensions of LAB and pathogenic bacteria (107 CFU/mL each) in MRS broth without sorbic acid were grown for 48-h and CFUs of bacteria were calculated on differential media. Data are shown as medians with IQR. Asterisks denote statistically significant difference with monocultures of pathogenic bacteria (*p* < 0.05)

When the pathogens and LAB strains were inoculated together in the MRS broth at equal cell densities to simulate the contamination and incubated for 48 h, pronounced repression of *S. aureus* and *E. coli* could be observed (Fig. 2). Moreover, the AG16 strain significantly repressed also the growth and the biofilm formation by *B. cereus*. In marked contrast, no significant repression of *P. aeruginosa* by any of the LAB could be observed despite the CFUs number of LAB in all mixed cultures remained within the range of 10^6^–10^10^ CFU/mL (Additional file 1: Fig. S2).

### The acidification rate

Among many tools of LAB providing their antagonistic properties against other bacteria, the synthesis of various organic acids leading to the acidification of the broth and repression of growth of other bacteria appears the keynote factor. The acidification rate was examined after 24 h growth in MRS broth in monoculture as well as after addition to 48-h old biofilms pre-formed by pathogenic bacteria (Table 2). In monocultures, all six LAB strains reduced the pH values by 3.2–3.6 units of standard pH scale and the total titratable acidity (TTA) of the culture liquid increased up to 1–2 mL in comparison with 0.5 mL in the initial broth. When LAB suspension in MRS have been added to the pre-established bacterial biofilms, a considerable reduction of the pH decrease could be observed in plates containing *E. coli* or *P. aeruginosa*, while no significant changes in comparison with LAB monocultures have been detected in the wells containing either *S. aureus* or *B. cereus* biofilms. Similarly, in the presence of either *S. aureus* or *B. cereus* biofilms the TTA increased up to 1.3–1.5 mL that was significantly higher in comparison with the probiotic strain *L. plantarum* 8PA3. On the contrary, in the plates containing *E. coli* or *P. aeruginosa* biofilms the TTA changed by 0.3–0.6 mL only.

**Table 2:** The broth^**‡**^ acidification rate by novel LAB strains isolated from silage growing in wells with 48-h old biofilms of pathogenic bacteria

	Glucose	Strains	*L. plantarum* AG 1	*P. acidilactici* AG 8	*L. plantarum* AG 9	*L. plantarum* AG 10	*L. plantarum* AG 15	*L. fermentum* AG 16	*L. plantarum* 8PA3
ΔpH	2%	*LAB monoculture*	3.4 ± 0.35*	3.2 ± 0.24*	3.4 ± 0.31*	3.6 ± 0.56*	3.3 ± 0.23*	3.2 ± 0.11*	2.3 ± 0.15
		*P. aeruginosa*	2.5 ± 0.55	2.6 ± 0.22*	2.5 ± 0.54	2.5 ± 0.5	2.4 ± 0.39	3.1 ± 0.91*	2.4 ± 0.51
		*S. aureus*	3.1 ± 0.21*	3.1 ± 0.14*	3.1 ± 0.21*	3.1 ± 0.23*	3.1 ± 0.23*	3.2 ± 0.25*	2.8 ± 0.25
		*E. coli*	2.0 ± 0.11	2.0 ± 1.08	2.0 ± 1.10	2.0 ± 1.15	2.0 ± 1.15	1.8 ± 1.44	1.8 ± 1.11
		*B. cereus*	3.3 ± 0.18*	3.4 ± 0.23*	3.2 ± 0.25*	3.1 ± 0.06*	3.1 ± 0.09	3.4 ± 0.50*	2.9 ± 0.41
	0.2%	*LAB monoculture*	2.5 ± 0.34*	2.0 ± 0.53*	2.2 ± 0.46*	1.8 ± 0.27*	2.2 ± 0.38*	2.2 ± 0.62*	1.2 ± 0.25
		*P. aeruginosa*	0.8 ± 0.79	1.0 ± 0.76*	0.2 ± 0.38	0.1 ± 0.88	1.0 ± 0.86*	1.0 ± 0.88*	0.7 ± 0.43
		*S. aureus*	1.7 ± 0.64*	1.6 ± 0.47*	1.7 ± 0.52*	1.7 ± 0.66*	1.7 ± 0.64*	1.6 ± 0.54*	0.6 ± 0.30
		*E. coli*	0.5 ± 0.68	0.1 ± 0.50	0.4 ± 0.37	0.5 ± 0.64*	0.6 ± 0.80*	0.3 ± 0.56	0.2 ± 0.62
		*B. cereus*	0	0	0	0	0	0	0
ΔTTA	2%	*LAB monoculture*	2.2 ± 0.63*	1.5 ± 0.43*	1.7 ± 0.65*	2.3 ± 0.87*	1.8 ± 0.57*	1.3 ± 0.23*	1.1 ± 0.01
		*P. aeruginosa*	0.3 ± 0.12	0.3 ± 0.10	0.3 ± 0.09	0.3 ± 0.05	0.3 ± 0.07	0.6 ± 0.03*	0.3 ± 0.04
		*S. aureus*	1.1 ± 0.21*	1.3 ± 0.18*	1.5 ± 0.65*	1.1 ± 0.21*	1.1 ± 0.21*	1.3 ± 0.34*	0.5 ± 0.02
		*E. coli*	0.7 ± 0.20	0.5 ± 0.15	0.5 ± 0.08	0.6 ± 0.09	0.6 ± 0.06	0.6 ± 0.06	0.6 ± 0.01
		*B. cereus*	1.3 ± 0.25*	1.3 ± 0.31*	1.5 ± 0.74*	1.3 ± 0.21*	1.4 ± 0.21*	1.4 ± 0.76*	0.7 ± 0.03
	0.2%	*LAB monoculture*	0.31 ± 0.11	0.30 ± 0.10	0.31 ± 0.08	0.30 ± 0.05	0.27 ± 0.03	0.25 ± 0.05	0.31 ± 0.06
		*P. aeruginosa*	0.03 ± 0.01	0.03 ± 0.01	0.05 ± 0.02	0.03 ± 0.01	0.02 ± 0.01	0.03 ± 0.02	0.01 ± 0.02
		*S. aureus*	0.05 ± 0.01	0.01 ± 0.01	0.05 ± 0.01	0.02 ± 0.02	0.05 ± 0.03	0.05 ± 0.02	0.02 ± 0.01
		*E. coli*	0.08 ± 0.01	0.50 ± 0.01	0.52 ± 0.01	0.5 ± 0.02	0.07 ± 0.02	0.23 ± 0.02	0.28 ± 0.09
		*B. cereus*	0.02 ± 0.01	0.05 ± 0.01	0.05 ± 0.01	0.03 ± 0.01	0.05 ± 0.02	0.05 ± 0.01	0.01 ± 0.02

^**‡**^ Suspensions of LAB (10^7^ CFU/mL) in MRS broth without sorbic acid were added into the wells with 48-h old biofilms pre-formed by pathogenic bacteria and incubation was continued for the next 24 h. ΔpH and ΔTTA were calculated as a difference between initial and final values. The Total Titratable Acidity (TTA) is expressed as mL of 0.1 M NaOH as required to achieve the final pH of 8.2

* denotes statistically significant difference with probiotic strain *L. plantarum* 8PA3 (*p* < 0.05)

In the experiments with simultaneous inoculation of LAB with *B. cereus, E. coli* or *S. aureus* their acidification activity was comparable with the corresponding monocultures level (Table 3) leading to the repression of growth and biofilm formation by *E. coli* and *S. aureus*, although less efficient (Fig. 2). By contrast, no acidification could be observed in the *P. aeruginosa*-LAB mixed cultures (Table 3) while viable LAB were identified in both culture liquid and biofilm, although the growth of AG8, AG15 and AG16 has been significantly repressed. Interestingly, no repression of AG9 and AG10 could be observed suggesting that *P. aeruginosa* did not repress their growth while neutralized the acid produced by them.

**Table 3:** The broth^**‡**^ acidification rate in mixed cultures of novel LAB strains and pathogenic bacteria

	Glucose	Strains	*L. plantarum* AG 1	*P. acidilactici* AG 8	*L. plantarum* AG 9	*L. plantarum* AG 10	*L. plantarum* AG 15	*L. fermentum* AG 16	*L. plantarum* 8PA3
ΔpH	2%	*LAB monoculture*	3.4 ± 0.35*	3.2 ± 0.24*	3.4 ± 0.31*	3.6 ± 0.56*	3.3 ± 0.23*	3.2 ± 0.11*	2.3 ± 0.15
		*P. aeruginosa*	0.5 ± 0.27*	0.5 ± 0.67*	0.6 ± 0.35*	0.4 ± 0.19*	0.5 ± 0.5*	0.8 ± 0.52*	−0.3 ± 0.29
		*S. aureus*	3.0 ± 0.62	3.0 ± 0.50	3.1 ± 0.52	3.0 ± 0.62	3.1 ± 0.51	3.1 ± 0.62	2.9 ± 0.23
		*E. coli*	2.6 ± 0.66	2.6 ± 0.75	2.6 ± 0.72	2.6 ± 0.65	2.6 ± 0.64	2.6 ± 0.61	2.3 ± 0.68
		*B. cereus*	3.6 ± 0.06*	3.8 ± 0.31*	3.7 ± 0.53*	3.7 ± 0.52*	3.7 ± 0.67*	4.0 ± 0.25*	2.9 ± 1.53
	0.2%	*LAB monoculture*	3.5 ± 0.34*	2.0 ± 0.53*	2.2 ± 0.46*	1.8 ± 0.27*	2.2 ± 0.38*	2.2 ± 0.62*	1.2 ± 0.25
		*P. aeruginosa*	−0.3 ± 0.16	− 0.3 ± 0.15	−0.3 ± 0.02	− 0.2 ± 0.01	−0.3 ± 0.13	− 0.2 ± 0.37	−0.3 ± 0.11
		*S. aureus*	1.2 ± 0.28	1.2 ± 0.38	1.3 ± 0.17	1.1 ± 0.33	1.3 ± 0.05	0.9 ± 0.16	1.2 ± 0.28
		*E. coli*	0.5 ± 0.69	0.3 ± 0.42	1.0 ± 0.56	0.6 ± 1.16	0.9 ± 0.15	0.3 ± 0.37	0.3 ± 0.97
		*B. cereus*	0	0	0	0	0	0	0
ΔTTA	2%	*LAB monoculture*	2.2 ± 0.63*	1.5 ± 0.43*	1.7 ± 0.65*	2.3 ± 0.87*	1.8 ± 0.57*	1.3 ± 0.23*	0.1 ± 0.01
		*P. aeruginosa*	0.9 ± 0.12*	0.6 ± 0.09	0.8 ± 0.08*	0.6 ± 0.34	0.7 ± 0.37*	0.7 ± 0.36*	0.1 ± 0.02
		*S. aureus*	1.1 ± 0.23	1.3 ± 0.16	1.5 ± 0.31	0.8 ± 0.42	1.4 ± 0.48	1.1 ± 0.39	1.5 ± 0.15
		*E. coli*	1.3 ± 0.22*	1.0 ± 0.11	1.1 ± 0.13	1.3 ± 0.61*	1.9 ± 0.13*	1.5 ± 0.35	0.8 ± 0.13
		*B. cereus*	1.7 ± 0.41*	1.5 ± 0.21	2.0 ± 0.56*	1.7 ± 0.24*	1.8 ± 0.28*	2.0 ± 0.29*	1.0 ± 0.08
	0.2%	*LAB monoculture*	0.31 ± 0.11	0.27 ± 0.10	0.30 ± 0.02	0.32 ± 0.05	0.27 ± 0.03	0.25 ± 0.05	0.31 ± 0.06
		*P. aeruginosa*	0.04 ± 0.02	0.04 ± 0.02	0.01 ± 0.01	0.01 ± 0.01	0.04 ± 0.02	0.04 ± 0.02	0.04 ± 0.02
		*S. aureus*	0.00 ± 0.01	0.00 ± 0.01	0.00 ± 0.01	0.00 ± 0.01	0.00 ± 0.01	0.00 ± 0.01	0.05 ± 0.01
		*E. coli*	0.05 ± 0.01	0.00 ± 0.01	0.11 ± 0.01	0.05 ± 0.01	0.05 ± 0.01	0.00 ± 0.01	0.05 ± 0.01
		*B. cereus*	0.31 ± 0.05	0.30 ± 0.05	0.31 ± 0.04	0.30 ± 0.05	0.31 ± 0.05	0.31 ± 0.05	0.29 ± 0.05

^**‡**^ Mixed suspensions of LAB and pathogenic bacteria (10^7^ CFU/mL each) in MRS broth without sorbic acid were grown for 48 h. ΔpH and ΔTTA were calculated as a difference between initial and final values. Total Titratable Acidity (TTA) is expressed as mL of 0.1 M NaOH needed to achieve the final pH of 8.2

* denotes statistically significant difference with probiotic strain *L. plantarum* 8PA3 (*p* < 0.05)

**Fig. 3:**
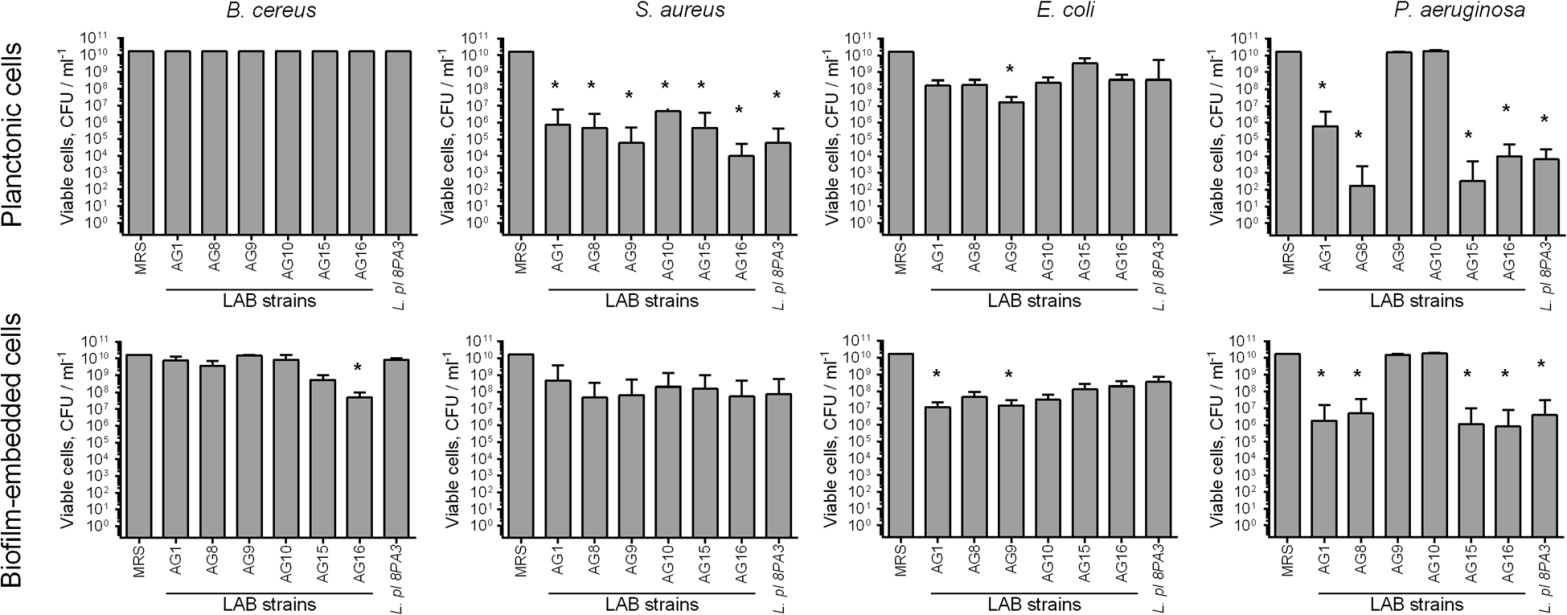
Antimicrobial activity of novel LAB strains against planktonic and biofilm embedded forms of pathogenic bacteria when growing in MRS with 10-fold reduced glucose content (0.2%). LAB suspensions in MRS broth (107 CFU/ml) were added to 48-h old biofilms of pathogenic bacteria. After 24 h CFUs of bacteria were calculated on differential media. Data are shown as medians with IQR. Asterisks denote statistically significant difference with monocultures of pathogenic bacteria (*p* < 0.05)

**Fig. 4:**
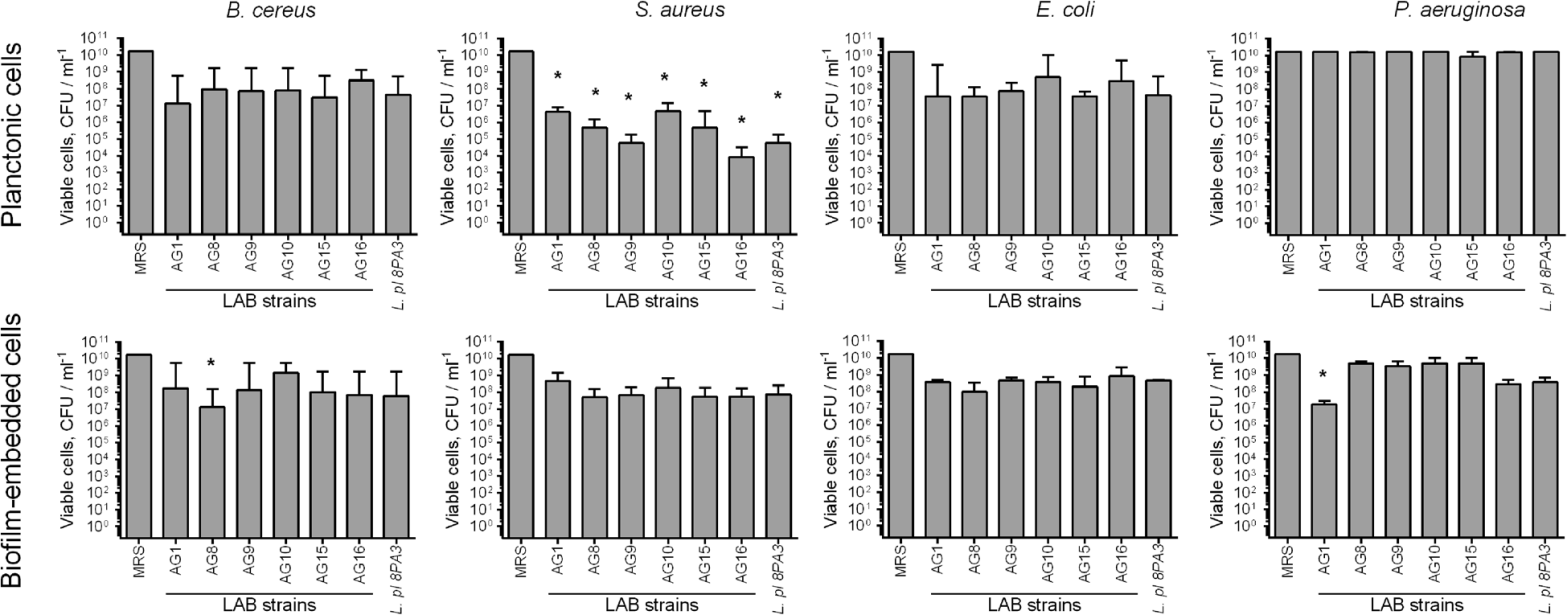
Repression of pathogenic bacteria by novel LAB in co-cultivation experiments in MRS with 10-fold reduced glucose content (0.2%). Suspensions of LAB and pathogenic bacteria (107 CFU/mL each) in MRS broth without sorbic acid were grown for 48-h and CFUs of bacteria were calculated on differential media. Data are shown as medians with IQR. Asterisks denote statistically significant difference with monocultures of pathogenic bacteria (*p* < 0.05)

Next, to check the significance of the acidification contribution to the antibacterial activity of LAB isolates, their antibacterial activity has been studied when growing in MRS with reduced amount of glucose (0.2%), which is the main substrate for the lactate production. Due to the lowered acidification rate in MRS containing 0.2% glucose in comparison with broth supplemented with 2% glucose (see Table 2), the antibacterial activity of LAB decreased (Fig. 3), while the cultures reached similar CFUs densities like in the full broth (compare Additional file 1: Fig. S1 and S3). These data suggest that the broth acidification seems to be the dominating antagonistic factor of both newly isolated and reference LAB strains. In contrast, strains AG8 and AG15 repressed the spreading of *P. aeruginosa* into the culture liquid from the biofilm independently of the glucose content (compare Figs. 1 and 3), although exhibiting slightly lower efficacy in low-glucose medium, suggesting the potential involvement of other antagonistic factors by these strains such as bacteriocines or hydrogen peroxide. When both LAB and pathogens were inoculated simultaneously in MRS containing 0.2% glucose, significant repression of only *S. aureus* growth could be observed (Fig. 4), most likely, due to low acidification rate (See Table 3).

### Strains identification

All six isolates demonstrated better growth capabilities at 37 °C compared to 30 °C, while remained capable of growing at temperatures up to 45 °C, were Gram-positive and catalase-negative. Five of them were rod-shaped and based on the 16S rRNA gene sequence were identified as *Lactobacillus plantarum* (AG1, AG9, AG10 and AG15, 99% identity) and *Lactobacillus fermentum* (AG8, AG16, 99% identity). At the same time the comparison of 16S rRNA genes of isolates among themselves did not reveal 100% identity, suggesting these isolates as different strains in agreement with antibacterial activity data.

### Tolerances to acid and bile

The ability to survive under gastric conditions including low pH and bile presence is an important property of LAB strains used in the food industry while it is strongly species- and strain- dependent. The tolerance of isolates was investigated by exposing cells to gastric-simulated conditions (pH 2.0, 1% bile) for 2 h. Isolates AG8, AG9, AG10 and AG15 demonstrated remarkable resistance with the growth reduction by 4–8 times in comparison with the reference *L. plantarum* strain (Table 4). AG10 strain appeared the most resistant one so far.

**Table 4:** Characterization of probiotic properties of novel LAB strains isolated from silage

LAB species	Strain	Survival rate, %	Hydrophobicity		Auto-aggregation, %	
			% ^a^	Grade ^b^	4 h	24 h
*L. plantarum*	AG1	6.0 ± 3.70	18.4 ± 2.40	L	26.8 ± 4.63	90.3 ± 7.83
*P. acidilactici*	AG8	13.1 ± 1.47	24.4 ± 2.00	L	26.0 ± 5.86	79.6 ± 8.98
*L. plantarum*	AG9	11.4 ± 0.60	55.6 ± 1.71	M	35.6 ± 3.04	88.7 ± 5.28
*L. plantarum*	AG10	26.0 ± 0.14	65.7 ± 7.52	M	21.7 ± 7.00	66.6 ± 7.44
*L. plantarum*	AG15	25.9 ± 0.70	3.2 ± 0.42	L	25.1 ± 6.52	78.5 ± 7.26
*L. fermentum*	AG16	3.5 ± 0.54	41.2 ± 5.06	M	26.4 ± 4.18	85.5 ± 5.40
*L. plantarum*	8PA3	8.2 ± 0.92	23.2 ± 1.03	L	23.1 ± 4.23	72.7 ± 8.35

^a^ Percentage of hydrophobicity

^b^ Strains were classified as low (L) or medium (M) according to their hydrophobicity capacities according to [44]

### Adhesion capacity

Percentages of autoaggregation after 4 and 24 h of incubation at 37 °C and cell surface hydrophobicity are summarized in Table 4. None of the strains demonstrated high hydrophobicity measured as their ability to adhere to hexadecane, while strains AG9, AG10 and AG16 showed medium hydrophobicity in comparison with other isolates and with the reference strain. After 4 h of incubation similar percentage of autoaggregation ranging from 21 to 27% was observed for all isolates with the exception of the AG9 strain (36% of autoaggregation). After 24 h low autoaggregation was detected for the strains AG8, AG10 and AG15, while for the strains AG1, AG9 and AG16 about 90% of autoaggregation could be observed.

### Antibiotic resistance

Table 5 summarizes the LAB isolates resistance against conventional antibiotics. In general, all LAB strains were resistant to streptomycin, aminoglycosides, vancomycin and ciprofloxacin. Taking into account high similarities in resistance patterns, we hypothesize that the tolerance to antimicrobials is governed rather by phenotypic resistance than by the presence of particular resistance genes and thus is likely non-transmissible.

**Table 5:** Antibiotic resistance of newly isolated LAB strains from silage (diameters of inhibition zones, mm)

Antibiotics	Amount per disc, μg	LAB strains					
		AG1	AG8	AG9	AG10	AG15	AG16
Ampicillin	10	26(S)	18(S)	20(S)	19(S)	20(S)	17(S)
Amikacin	30	12(R)	16(I)	9(R)	10(R)	3(R)	8(R)
Chloramphenicol	30	22(S)	25(S)	28(S)	23(S)	22(S)	29(S)
Ciprofloxacin	5	0(R)	0(R)	0(R)	0(R)	0(R)	9(R)
Clindamycin	2	16(S)	15(S)	33(S)	18(S)	28(S)	24(S)
Erythromycin	15	20(S)	20(S)	26(S)	22(S)	20(S)	26(S)
Gentamicin	10	10(R)	11(R)	10(R)	9(R)	0(R)	13(S)
Kanamycin	30	0(R)	0(R)	6(R)	0(R)	0(R)	5(R)
Rifampicin	5	20(S)	26(S)	20(S)	20(S)	13(R)	30(S)
Streptomycin	30	10(R)	14(I)	8(R)	11(R)	11(R)	11(R)
Tetracycline	30	20(S)	20(S)	20(S)	21(S)	19(S)	25(S)
Vancomycin	30	0(R)	0(R)	6(R)	14(R)	0(R)	2(R)

Inhibition zones (mm) (means ± SD of 3 trials) were interpreted as susceptible (S), intermediate (I), or resistant (R) according to [45]

### Fermentation properties of selected LAB strains in skimmed milk

All six newly isolated LAB successfully demonstrated their ability of skimmed milk fermentation (Table 6). In general, the keynote properties of the products obtained by these strains were comparable with the yoghurt obtained by using *L. bulgaricus* and *S. thermophilus* mixture as a starter culture. In particular, the contents of total protein, whey protein and lactose were comparable in all obtained products. The fat concentration remained unchanged during the fermentation and remained at 0.2%. While in all cases pH dropped from 6.7 to 3.6–3.9, the highest acidification rate was observed for the AG8 and AG10 strains (Table 6), which also exhibited pronounced antibacterial activity (see Table 1 and Fig. 1). Moreover, these two strains provided the lowest syneresis in products (19%) that is significantly lower than in the classical yoghurt. Furthermore, the water hold capacity (WHC) of the products obtained with AG8, AG10 and AG15 strains was similar to that of the classical yoghurt. These data allows suggesting these strains as perspective cultures for co-introduction into LAB pre-cultures for milk fermentation. In marked contrast, using AG1, AG9 and AG16 strains resulted in products with lower WHC and higher syneresis suggesting lower quality of the final product.

**Table 6:** Properties of fermented skimmed milk

Strain	Protein, %	Whey protein, %	Lactose, %	Solids, %	pH	TTA. ml	WHC^a^, %	Syneresis, g water/100 g yoghurt	Dynamic viscosity, sec
AG1	4.1 ± 0.08	3.0 ± 0.11	4.5 ± 0.07	8.8 ± 0.11	3.7 ± 0.08	1.0 ± 0.01	36 ± 4.2	22 ± 2.9	6.6 ± 0.17
AG8	4.2 ± 0.10	3.1 ± 0.08	4.5 ± 0.11	9.4 ± 0.14	3.7 ± 0.08	1.1 ± 0.02	37 ± 1.60	19 ± 1.5	7.1 ± 0.09
AG9	4.2 ± 0.08	3.0 ± 0.11	4.5 ± 0.08	9.4 ± 0.20	3.7 ± 0.20	1.0 ± 0.02	35 ± 2.88	24 ± 3.5	7.8 ± 0.28
AG10	4.1 ± 0.08	3.1 ± 0.08	4.6 ± 0.04	9.5 ± 0.17	3.6 ± 0.08	1.1 ± 0.02	36 ± 2.88	19 ± 4.2	7.1 ± 0.17
AG15	4.1 ± 0.10	3.1 ± 0.06	4.4 ± 0.14	9.7 ± 0.07	3.9 ± 0.13	1.0 ± 0.02	37 ± 3.48	24 ± 3.1	6.0 ± 0.22
AG16	4.2 ± 0.10	3.0 ± 0.06	4.5 ± 0.10	9.1 ± 0.09	3.9 ± 0.08	1.0 ± 0.01	34 ± 2.11	24 ± 2.1	6.6 ± 0.11
Yogurt	4.2 ± 0.22	3.1 ± 0.04	4.5 ± 0.11	11.1 ± 0.11	3.9 ± 0.21	0.9 ± 0.02	39 ± 3.48	23 ± 3.5	7.2 ± 0.17
Milk	4.2 ± 0.09	–	4.7 ± 0.08	8.7 ± 0.173	6.7 ± 0.10	0.2 ± 0.01	–	–	–

^a^*WHC* Water holding capacity

## Discussion

In this work we report the newly isolated LAB strains from clover silage exhibiting antibacterial activity as well as attractive probiotic and milk-fermenting properties. We particularly aim to identify bacterial strains with high antibacterial activity against biofilm-embedded pathogens as they often exhibit pronounced resistance against conventional antimicrobials and thus possess a serious threat for the food industry where the use of biocides is strictly limited. In contrast, being used either as new starter cultures for the milk fermentation or as minor additive components to the classical cultures, LAB strains characterized by significant antagonism against biofilm-embedded pathogenic bacteria could prevent product contamination in the first place thus reducing the need in often highly toxic biocides that is altogether in line with the development of green food technologies.

During the initial screening, six strains with the highest antimicrobial activity have been selected (Table 1), and five of them (excepting AG15) were able to efficiently eradicate the biofilms of pathogenic *S. aureus* and *E. coli* and prevent their spreading from the biofilm by either being added to preformed biofilms or prevent the growth of bacteria in co-cultivation experiments (Figs. 1 and 2). While no repression of *P. aeruginosa* could be observed during co-cultivation, AG8, AG10 and AG16 strains, identified as *L. plantarum* and *L. fermentum*, were able to eradicate this pathogen in the biofilm almost completely and repress to a certain extent its spreading from the biofilm into the culture liquid (Fig. 1). Moreover, in co-cultivation experiments AG16 also repressed the biofilm formation and the growth of *B. cereus* (Fig. 2) being thereby an attractive tool for the prevention of food contamination by this pathogen, which forms the biofilm with high resistance to many conventional antimicrobials [46].

The base of antimicrobial activities of these strains is likely linked with the acidification of the growth media, since the reduction of glucose from 2 to 0.2% with consequent drastic decrease of acidification rate (Table 3) led to significantly reduced antagonistic properties of the strains (Fig. 3). Therefore the absence of the *P. aeruginosa* growth repression in co-cultivation experiments could be assumed as a consequence of no broth acidification in the mixed culture (Table 2). Of note, the activity of AG8 and AG15 strains against *P. aeruginosa* biofilms did not depend of glucose content, suggesting the production of other antagonistic factors by these strains, possibly, bacteriocines or hydrogen peroxide.

The investigations of other probiotic properties of the novel LAB isolates revealed that the strain AG10 exhibits the best characteristics. It demonstrated the highest hydrophobicity on hexadecane (66%), medium auto-aggregation rate (67% in 24 h) and appeared the most resistant to simulated gastric conditions (Table 4) among all tested strains. Since the hydrophobicity and autoaggregation properties are believed to be associated with the adhesion of LAB to epithelial cells in human intestine that is required for their probiotic properties [47, 48] and the hydrophobicity against hexadecane has been reported to be within the range of 5–50%, our data suggests pronounced adhesion properties of the AG10 strain. Moreover, the milk fermented by AG10 was characterized by low syneresis, high WHC and highest acidification (Table 6). These data allows suggesting *Lactobacillus plantarum* AG10 strain as a potential starter culture and/or functional component for the production of fermented milk and yoghurt additionally characterized by pronounced anti-biofilm properties.

## Conclusion

Among the six novel LAB strains isolated from the silage and exhibiting high acidification rate and pronounced antagonism with various both planktonic and biofilm-embedded foodborne pathogenic bacteria, the *Lactobacillus plantarum* strain AG10 demonstrated beneficial probiotic and milk fermentation properties while remaining resistant to simulated gastric conditions. AG10 efficiently eradicates biofilms of foodborne pathogens including *S. aureus*, *E. coli* and *P. aeruginosa* and prevents spreading of pathogens from the biofilm, as well as demonstrates attractive probiotic properties. These data allows suggesting this strain as perspective starter culture and/or functional component for the fermented milk.

## Methods

### Isolation of LAB and growth conditions

Lactic acid bacteria were isolated from 5-month fermented silage (“Zavolzh’e”, Kaibitsy district, Republic of Tatarstan, Russia). Silage samples (10 g) were blended with 50 ml of sterile saline (0.9% NaCl) and after intensive shaking a series of 10-fold dilutions in saline were prepared (10^− 1^ – 10^− 8^). Each dilution (1 ml) was mixed with 20 ml of cabbage agar (CA) (cabbage 200 g, glucose 20 g, peptone 10 g, agar 20 g, water 1000 ml) containing 3% CaCO3 and plated. The plates were incubated under microaerophilic conditions at 37 °C for 48 h. The bacterial colonies forming clear zones of CaCO_3_ hydrolysis were considered as putative LAB and were individually picked and streaked on de Man, Rogosa and Sharpe (MRS) agar (HiMedia, India) plates by dilution streaking. The plates were incubated under microaerophilic conditions at 37 °C until the single colonies were obtained; these isolates were then subjected to Gram staining and catalase test. Only catalase-negative and Gram-positive isolates were selected for further studies. Bacterial isolates were maintained on solid MRS agar for immediate use or in 50% glycerol at − 80 °C.

### Reference probiotic LAB and pathogenic bacteria

*Lactobacillus plantarum* 8PA3 approved as a probiotic strain (Biomed, Russia) was used as a reference [49]. A mixture of *Streptococcus thermophilus* and *Lactobacillus delbrueckii* ssp. *bulgaricus* (CFUs ratio 1:1) was used as a reference classic yoghurt starter (Yoghurtel, Russia). *Escherichia coli* MG1655 (K-12), *Staphylococcus aureus ssp. aureus* ATCC 29213, *Klebsiella pneumonia* (clinical isolate)*, Pseudomonas aeruginosa* АТСС 27853, and *Bacillus cereus* (clinical isolate) were used in this study as test bacteria when evaluating the antibacterial activity of LAB. Clinical isolates of *B. cereus* and *K. pneumoniae* were kindly provided by Kazan Institute of Epidemiology and Microbiology (Kazan, Russia) and by the Institute of Medical Microbiology (Giessen, Germany), respectively.

### Antibacterial activity

Antagonistic activity was examined by agar spot test described in [50]. Briefly, overnight cultures of individual strains were spotted (2 μl) on the surface of MRS agar and incubated anaerobically (Anaerogas gaspack, NIKI MLT, Russia) for 24 h at 37 °C to develop the spots. A 100-μl volume of an overnight culture of test bacteria was mixed with 7 ml of soft Luria-Bertani (LB) agar (0.7%), poured over the plate and plates were incubated aerobically at 37 °C. After 24 h of incubation, zones of bacterial growth inhibition were measured from the edge of the colony to the edge of the inhibition zone. The inhibitory effect of MRS was used as negative control. Each test was performed in triplicate.

Additionally, the antagonism of LAB strains was tested against pathogens embedded into the biofilm. For that, 48-h old biofilms of pathogenic bacteria were grown on 24-well polystirol plates in BM broth [51–53], washed with sterile saline (0.9% NaCl) and wells were filled with MRS broth without sorbic acid containing 10^7^ CFU/ml of LAB isolates (diluted 24-h old culture); *L. plantarum* 8PA3 was used as a reference strain. As a control, a cell-free MRS broth was used. CFUs were counted by using the drop-plate assay [54] with modifications [53, 55]. After 24 h, 10-fold dilution series of culture liquid and the suspended biofilm were prepared and plated on differential media by drops (5 μL each). LAB were plated onto MRS agar, mannitol-salt agar (Sigma) was used for *S. aureus*, Endo agar (Sigma) was used for *E. coli*, cetrimide agar (Sigma) was used for *P. aeruginosa*. *B. cereus* cells were seeded on LB agar and bigger colonies with rough surfaces have been considered as *B. cereus*. CFUs were counted from the two last drops typically containing 5–15 colonies. Data from 5 independent experiments were presented as medians with IQR.

### Acidification rate

The acidification rate of the strains was evaluated by measuring pH and Total Titratable Acidity (TTA) of the culture liquid after 24 h growth in MRS broth. An overnight culture (1% v/v) of each LAB strain was individually inoculated into 5 ml of MRS broth and incubated for 24 h at 37 °C. Then the pH and TTA of the culture liquid were measured. To determine TTA, the cell-free supernatant (1 mL) was titrated with 0.1 M NaOH to a final pH of 8.2, detected by phenolphthalein; TTA was expressed as mL of 0.1 M NaOH needed to achieve the final pH of 8.2. The cell-free broth incubated together with other samples served as a reference.

### Tolerance to simulated human GI tract (acid and bile tolerance)

Synthetic gastric fluid was prepared by suspending 8.3 g of proteose peptone, 3.5 g D-glucose, 2.05 g NaCl, 0.6 g KH_2_PO_4_, 0.11 g CaCl_2_, 0.37 g KCl, 0.05 g bile, 0.1 g lysozyme, 13.3 mg pepsin in 100 ml sterile distilled water. pH value was adjusted to 2.5 with 1 N HCl [56]. Overnight LAB cultures were harvested by centrifugation and washed twice with physiological saline. Cell suspensions were adjusted to OD_600_ of 0.5 and treated with synthetic gastric fluid at 37 °C for 1 h. As a control, cells were incubated in physiological saline at 37 °C for 1 h. After the incubations, the cells were washed twice with physiological saline by centrifugation, stained with 2.5 μg/ml propidium iodide (PI) (Sigma-Aldrich) and analyzed by flow cytometry using a BD FACS Canto II (USA) flow cytometer. PI enables staining of non-viable cells. Data were obtained using FACS Diva software. Survival rate (%) was calculated as: N_1_ / N_0_ × 100, where N_1_ represents the count of cells non-stained with PI in test sample and N_0_ represents the total count of cells non-stained with PI in the control sample.

### Cell surface hydrophobicity

The bacterial adhesion to hexadecane was measured as described in [57]. The bacterial cells grown in the MRS broth at 37 °C for 18 h were centrifuged, the cell pellet was washed twice with 0.1 M KNO_3_ (pH 6.2) following by its resuspension in the same solution to an optical density of 0.4 at 400 nm (A_0_). Bacterial cell suspensions (2.4 ml) and n-hexadecane (0.4 ml) were mixed by vortexing and incubated at 37 °C for 15 min for a complete phase separation in the mixture. The aqueous phase was gently taken out to measure its absorbance at 400 nm (A_1_). The surface hydrophobicity (%) was calculated as (1 - A_1_/A_0_) × 100. Strains were classified as low (L) or medium (M) according to their hydrophobicity capacities [44].

### Autoaggregation

Autoaggregation ability of the isolates was tested following the methodology described in [58]. Briefly, the bacterial cells grown in MRS broth at 37 °C for 18 h were centrifuged, the cell pellet was washed twice with PBS and then resuspended in PBS to an optical density of 0.5 at 600 nm (A_0_). Bacterial cell suspensions (4 ml) were incubated at 37 °C in tubes for 4 or 24 h without shaking. The aqueous phase was gently taken out to measure its absorbance at 600 nm (A_1_). Autoaggregation percentage was calculated as (1 – A_1_/A_0_) × 100.

### Resistance to antibiotics

Antibiotic susceptibility of the LAB isolates was determined by disc diffusion method, as described earlier [59]. Briefly, bacteria were grown in MRS broth overnight at 37 °C in anaerobic conditions (Anaerogas gaspack, NIKI MLT, Russia), and pour-plated on MRS agar plates. Antibiotic discs (Scientific Research Centre of Pharmacotherapy, Russia) were placed on the surface of inoculated plates. All isolates were screened for their susceptibility to ampicillin (10 μg), amikacin (30 μg), vancomycin (30 μg), gentamicin (10 μg), kanamycin (30 μg), clindamycin (2 μg), rifampicin (5 μg), streptomycin (30 μg), tetracycline (30 μg), erythromycin (15 μg), ciprofloxacin (5 μg), chloramphenicol (30 μg). After 48 h incubation in anaerobic conditions at 37 °C, the diameter of the inhibition zone was measured and interpreted as susceptible (S), intermediate (I), or resistant (R) according to [45]. For ampicillin (≤16 mm) and rifampicin (≤16 mm), breakpoints recommended for *Enterococcus* spp. were used [60].

### Genomic DNA isolation

Genomic DNA was isolated from cells grown at 37 °C for 16–18 h in MRS broth. Cells were harvested from 10 ml of culture liquid by centrifugation followed by resuspension in 1 ml of 50 mM Tris-HCl buffer (pH 8.0) containing lysozyme (3 mg/ml). After 2 h incubation at 37 °C, SDS was added until the final concentration of 1% was reached. After lysis of cells, 0.5 ml of phenol-chloroform mix was added and after intensive vortexing the mixture was separated by centrifugation at 14000 rpm for 5 min. This step was repeated for three times. 0.7 ml of upper fraction was mixed with 1 ml of propanol-2 and precipitated DNA was collected, washed by ethanol and dissolved in pure water.

### Identification of LAB isolates

The PCR reaction was carried out in a total volume of 25 μl by using universal 16S rRNA bacterial primers 27F (5′-GAGTTTGATCCTGGCTCAG-3′) and 1392R (5′- ACGGTTACCTTGTTACGACTT-3′) as offered by [61] and successfully used in [62, 63]. The DNA fragments were purified from the agarose gel after electrophoresis and sequenced on an ABI Prism 3730 sequencer (Applied Biosystems). For species identification, sequences were aligned with NCBI database using BLAST algorithm (https://www.ncbi.nlm.nih.gov/BLAST/).

### Experimentally fermented skimmed milk

Six selected strains that demonstrated promising properties during preliminary screening were used for experimental milk fermentation. To determine the fermentation capacity, pre-cultures of LAB were prepared by incubation at 40 °C for 16 h in skimmed milk obtained by raw cow’s milk pasteurization at 80 °C for 30 min followed by final centrifugation at 3000 g for 10 min. Obtained pre-cultures were inoculated (5% v/v) into the milk and incubated at 40 °C for 6 h followed by cooling for 24 h at 4 °C. In the following, a series of properties of the fermented milk have been analyzed.

### Quantitative chemical analysis of fermented skimmed milk

Analysis of protein, lactose and solids contents in the yogurt was performed on the InfraLUM® FT-12 (Russian Federation) with appropriate software and calibration data for the product “yoghurt”. Whey total protein was tested in supernatant of the fermented milk after centrifugation at 3000 g 15 min. To measure the acidification rate, a 2% w/v dispersion of the fermented milk was shaken in pure water for 5 min and the pH was determined. TTA was measured as described above by titration with NaOH in presence of phenolphthalein.

To determine the water holding capacity, 20 g of fermented milk (Y) after cooling to + 4 °C for 24 h was centrifuged for 10 min at 3000 rpm, the released whey (W) was removed and weighed. The Water-holding capacity (WHC) of fermented milk was calculated as WHC = (Y-W)/Y × 100%. To measure the syneresis centrifugation has been performed at 500 rpm for 5 min. The syneresis was expressed in grams of supernatant per 100 g of the product.

### Statistical analysis

All experiments were performed in biological triplicates with three repeats in each run. The data were analyzed and graphically visualized using GraphPad Prism version 6.00 for Windows (GraphPad Software, USA, www.graphpad.com). In each experiment, comparison against the reference strain has been performed using the non-parametric Kruskal–Wallis one-way analysis of variance test. For the drop-plate assays results assessed from 10-fold dilutions, where typically only in the two latter dilutions the number of colonies was countable, to assess the statistical significance, we compared 10 log_10_(*c*), where *c* is the obtained cell number, using the Pearson’s chi-squared homogeneity test. For both tests significant differences were reported at *p* < 0.05.

## Supplementary information

**Additional file 1: Figure S1.** Viability of LAB strains when growing for 24 h in presence of 48-h old biofilms of pathogenic bacteria. After 24 h CFUs of bacteria were calculated by seeding on MRS. Asterisks denote statistically significant difference with monocultures of corresponding LAB (*p* < 0.05). **Figure S2.** Viability of LAB strains during co-cultivation with pathogenic bacteria. After 48 h CFUs of bacteria were calculated by seeding on MRS. Asterisks denote statistically significant difference with monocultures of corresponding LAB (p < 0.05). **Figure S3.** Viability of LAB strains when growing in MRS with 10-fold reduced glucose content (0.2%) for 24 h in presence of 48-h old biofilms of pathogenic bacteria. After 24 h CFUs of bacteria were calculated by seeding on MRS. Asterisks denote statistically significant difference with monocultures of corresponding LAB (*p* < 0.05). **Figure S4.** Viability of LAB strains during co-cultivation with pathogenic bacteria in MRS with 10-fold reduced glucose content (0.2%). After 48 h CFUs of bacteria were calculated by seeding on MRS. Asterisks denote statistically significant difference with monocultures of corresponding LAB (*p* < 0.05).

## Data Availability

The datasets used and/or analyzed during the current study are available from the corresponding author on reasonable request.
