# Supplementary material for: Newly isolated lactic acid bacteria from silage targeting biofilms of foodborne pathogens during milk fermentation
Source: BMC Microbiol. 2019 Nov 8;19:248. doi: 10.1186/s12866-019-1618-0 (PMC6839075; doi:10.1186/s12866-019-1618-0)
Supplement: Supplementary file 1 — Additional file 1: Figure S1. Viability of LAB strains when growing for 24 h in presence of 48-h old biofilms of pathogenic bacteria. After 24 h CFUs of bacteria were calculated by seeding on MRS. Asterisks denote statistically significant difference with monocultures of corresponding LAB (p < 0.05). Figure S2. Viability of LAB strains during co-cultivation with pathogenic bacteria. After 48 h CFUs of bacteria were calculated by seeding on MRS. Asterisks denote statistically significant difference with monocultures of corresponding LAB (p < 0.05). Figure S3. Viability of LAB strains when growing in MRS with 10-fold reduced glucose content (0.2%) for 24 h in presence of 48-h old biofilms of pathogenic bacteria. After 24 h CFUs of bacteria were calculated by seeding on MRS. Asterisks denote statistically significant difference with monocultures of corresponding LAB (p < 0.05). Figure S4. Viability of LAB strains during co-cultivation with pathogenic bacteria in MRS with 10-fold reduced glucose content (0.2%). After 48 h CFUs of bacteria were calculated by seeding on MRS. Asterisks denote statistically significant difference with monocultures of corresponding LAB (p < 0.05). [file 12866_2019_1618_MOESM1_ESM.docx]

Novel lactic acid bacteria from silage for targeting the foodborne pathogens biofilms during milk fermentation

Elisaveta Gavrilova^1^, Elisaveta Anisimova^1^, Alsu Gabdelkhadieva^2^, Elena Nikitina^1, 2^, Adel Vafina^2^, Mikhail Bogachev^3^, Dina Yarullina^1^ and Airat Kayumov^1,^*

^1^ Kazan Federal University; info@kpfu.ru

^2^ Kazan National Research Technological University; [office@kstu.ru](mailto:office@kstu.ru)

^3^ Saint-Petersburg Electrotechnical University; [office@kstu.ru](mailto:office@kstu.ru)

***** Correspondence: kairatr@yandex.ru; Tel.: +7-843-233-78-02

**Fig. S1**. Viability of LAB strains when growing for 24 h in presence of 48-hours old biofilms of pathogenic bacteria. After 24 h CFUs of bacteria were calculated by seeding on MRS. Asterisks denote statistically significant difference with monocultures of corresponding LAB (p<0.05).

**Fig. S2**. Viability of LAB strains during co-cultivation with pathogenic bacteria. After 48 h CFUs of bacteria were calculated by seeding on MRS. Asterisks denote statistically significant difference with monocultures of corresponding LAB (p<0.05).

**Fig. S3**. Viability of LAB strains when growing in MRS with 10-fold reduced glucose content (0.2%) for 24 h in presence of 48-hours old biofilms of pathogenic bacteria. After 24 h CFUs of bacteria were calculated by seeding on MRS. Asterisks denote statistically significant difference with monocultures of corresponding LAB (p<0.05).

**Fig. S4**. Viability of LAB strains during co-cultivation with pathogenic bacteria in MRS with 10-fold reduced glucose content (0.2%). After 48 h CFUs of bacteria were calculated by seeding on MRS. Asterisks denote statistically significant difference with monocultures of corresponding LAB (p<0.05).
